# Incorporating mixed reality head mounted display technology in biportal endoscopic lumbar surgery: an early feasibility study

**DOI:** 10.3389/fsurg.2026.1772853

**Published:** 2026-03-05

**Authors:** Hana-Joy E. Hanks, Michael S. Kim, Rowen Lin, Vivan Chen, Andy T. Ton, Emily Mills, Hao-Hua Wu, Sohaib Z. Hashmi, Yu-Po Lee, Nitin N. Bhatia, Wongthawat Liawrungrueang, Max Meng-Huang Wu, Jung-Woo Hur, Don Young Park

**Affiliations:** 1 Irvine Department of Orthopaedic Surgery, University of California, Orange, CA, United States; 2 College of Osteopathic Medicine, Touro University Nevada, Henderson, NV, United States; 3 Department of Orthopaedic Surgery, University of Phayao, Phayao, Thailand; 4 Spine & Joints Center, Department of Orthopaedics, Samitivej Srinakarin Hospital, Bangkok, Thailand; 5 APSS-AOSpine AP Frontier Technologies Research Study Group, Taipei, Taiwan; 6 Department of Orthopaedic Surgery, Taipei Medical University, Taipei, Taiwan; 7 Eunpyeong St. Mary's Hospital, The Catholic University of Korea, Seoul, Korea

**Keywords:** Apple Vision Pro, biportal endoscopic spine surgery, clinical outcomes, endoscopic spine surgery, mixed reality

## Abstract

**Introduction:**

Mixed reality (MR) technology has emerged as a promising technology to endoscopic spine surgery by enhancing surgeon visualization. This early feasibility study introduces the Apple Vision Pro (Apple Inc., Cupertino, CA) Head Mounted Display (AVP HMD) as an intraoperative visualization tool during biportal endoscopic spine surgeries. The SURG-TLX is an established workload assessment tool specifically tailored for surgical procedures and is a specialized modification of the NASA-TLX, a widely established multidimensional measure for cognitive workload.

**Methods:**

Adult patients undergoing biportal endoscopic lumbar surgery using the AVP HMD were prospectively followed. SURG-TLX Scores were recorded immediately after each operation to document the cognitive workload of using the AVP HMD during surgery. Demographics, intraoperative, and postoperative complications were collected and assessed. Patient reported outcomes (PROs) were recorded with visual analogue scores (VAS) Back and Leg pain, as well as Oswestry Disability Index (ODI).

**Results:**

Forty patients were included in this study. Patients were followed for 3 months after surgery. The mean age of the population was 62.78 ± 16.12 years, with a BMI of 27.90 ± 5.86, with 47.5% being female. Preoperative average VAS Back score was 5.4 ± 3.26, VAS Leg scores was 6.85 ± 2.43, ODI score was 44% ± 18.67%. Average SURG-TLX score was 22.24 ± 7.46. There were 2 intraoperative dural tears with no clinical sequelae, otherwise there were no perioperative complications. At 3 months follow-up, the average post-operative VAS Back was 2.71 ± 3.29, VAS Leg was 2.11 ± 3.19, and ODI was 21.0% ± 22.74, which were significant reductions as compared to the preoperative scores (*p* < 0.05).

**Conclusion:**

This early feasibility study introduced the use of the AVP HMD during biportal endoscopic spine surgeries and showed that the AVP HDM did not increase the operating surgeon's perceived cognitive workload. The intraoperative use of AVP HMD did not worsen early clinical outcomes and did not increase the risk of complications. We describe the feasibility of incorporating MR technology such as the Apple Vision Pro for surgical visualization during endoscopic spine surgery.

## Introduction

Augmented reality (AR), virtual reality (VR), and mixed reality (MR) technology has recently been introduced in spine surgery (1, 2). AR superimposes digital images onto the real environment through the use of a smartphone, tablet, or glasses. VR completely immerses the user in a digital world using a headset that entirely blocks out the real environment. MR seamlessly merges real and digital environments together so that the user can interact with elements of both environments simultaneously using an immersive headset. These technologies have demonstrated utility in various aspects of healthcare including pre-operative planning, surgical simulation, and medical education (3).

Endoscopic spine surgery allows for direct visualization of the surgical anatomy with minimal disruption of the surrounding paraspinal musculature and soft tissues (4). Biportal endoscopic spine surgery utilizes a 4–5 mm incision for the viewing portal and a separate 8–10 mm incision for the working portal, allowing for increased versatility in the surgical field. Endoscopic camera technology can now display endoscopic video in 4 K, producing exceptional visualization of spinal anatomy. The natural next step of endoscopic spine surgery is incorporating MR technology by virtually overlaying 4 K video projections in large format, which can be precisely positioned by the surgeon, thus minimizing distractions from the line of vision and improving surgical precision (1, 5, 6).^.^ Previous studies have shown the feasible use of AR in spine surgery used in pedicle screw placement where AR-based navigation had comparable accuracy to more mainstream robotic-assisted methods (7–9).

Recently, the Apple Vision Pro (Apple Inc., Cupertino, CA), MR Head Mounted Displays (AVP HMD) was utilized during surgery to display live endoscopic video, pre-operative imaging, and patient clinical information simultaneously to minimize interruption of surgeon's line of sight from the operating field (5). The AVP HMD is an advanced MR system that integrates multiple 4 K outward cameras, eye tracking sensors, and 4 K displays to each eye within the headset, allowing for 4 K video capture of the real environment and precise spatial overlay of digital information on the real-world view for a seamless viewing experience (10, 11). As HMDs become increasingly ergonomic and computationally powerful, this technology is poised to expand its scope of practice in the field (5, 12).

Emerging literature suggests an increasing trend of AR/VR/MR integration in the operating room with potential for surgeon customization and intraoperative teaching (1–3). However, no prior clinical studies have investigated the direct impact on these technologies when utilized during endoscopic lumbar spine surgery. The Surgical Task Load Index (SURG-TLX) is a validated workload assessment tool specifically developed for surgical procedures and is a specialized modification of the NASA-TLX. NASA-TLX is a widely established multidimensional evaluation tool created by the NASA Ames Research Center to assess the perceived workload in machine and aviation, which has been cited in over 4,000 published studies (13–15). SURG-TLX is a modification of NASA-TLX to assess the task load of a surgical procedure using 6 six self-perceived domains: mental demand, physical demand, temporal demand, task complexity, situational stress, and distractions. SURG-TLX was used in this present study to evaluate the impact of the AVP HMD on the operating surgeon during endoscopic spine surgeries. In this study, we illustrate the feasibility of MR technology in endoscopic spine surgery.

## Methods

### Patient population

SURG-TLX scores were collected prospectively from a consecutive cohort of adult patients who underwent biportal endoscopic lumbar laminotomy, decompression, and/or discectomy while utilizing the AVP-HMD throughout the procedure. Institutional review board (IRB) approval was obtained (IRB protocol #: 5538). Patient inclusion began in March 2025 and continued to the three month follow up with patient-reported outcomes recorded and analyzed. All patients were treated by a single fellowship-trained spine surgeon who has performed over 700 biportal endoscopic spine surgeries. Patients were included based on the inclusion criteria: (1) biportal endoscopic lumbar laminotomy, decompression, and/or discectomy was performed using AVP HMD, (2) SURG-TLX Scores was obtained and recorded immediately after the completion of the surgery, (3) patient reported outcomes were obtained and recorded during post-operative visit. A total of forty patients were included in the final analysis.

The collected data included patient demographics including age, sex, BMI, and comorbidities. Pre-operative patient reported outcomes (PROs) of visual analogue scores (VAS) for both back and leg pain as well as Oswestry Disability Index (ODI) for functionality were compared to the post-operative values. Intraoperative data was collected including operative time, device use, complications (e.g., incidental durotomy).

### Workload questionnaire

The SURG-TLX is a common measurement tool of perceived cognitive load specifically designed for surgical tasks (16). The tool evaluates six dimensions of the procedure with: mental demand, physical demand, temporal demand, task complexity, situational stress, and distractions. Prior to evaluating the dimension ratings for each surgery, the surgeon is presented with every possible pair of the six dimensions with 15 pairs total. For each pair, the surgeon chooses which dimension contributed more to the perceived workload of that particular surgery. The number of times that each dimension is chosen is then counted to formulate the weight of the dimension for that specific case. A final score based on the following equation.

$$\mathrm{Final}\;\mathrm{score}\;=\sum (\mathrm{Weigh}t_{i}\times \mathrm{Ratin}g_{i})/15$$

(i) Each Dimension

The scoring scale is from a range of 0 to 100 with higher scores correlated with higher perceived mental workload (14–16).

### Surgical workflow and AR integration

The AVP HMD was utilized intraoperatively during biportal endoscopic lumbar spine procedures as previously described (5). Prior to the primary surgeon gowning and gloving, the digital endoscopic video was connected to the AVP HMD through an NDI encoder from the endoscopic video console by HDMI cable. The NDI encoder is then connected by ethernet cable to a separate Wi-Fi router solely dedicated to the AVP and optimized at the 6Ghz setting to ensure extremely fast, consistent, and reliable connection. This step is crucially important to minimize video lag experienced by the surgeon during surgery. The estimated latency range measured by the NDI analysis system was 50–70 ms, which is imperceptible to the human eye within this range. The headset was then donned and calibrated to the surgeon's visual field prior to scrubbing into the procedure. Donning of the headset can be completed prior to the start of the procedure since the MR device allows for real-world visualization with digital projections overlaying the real environment. The surgeon can place the digital 4 K video projection in any position in space and customize the size of the projection using the AVP hand gestures. The AVP uses hand gestures to control the virtual applications, such as enlarging and minimizing the application, recording video, and taking still pictures. Due to these technological properties, the AVP HMD can be used while maintaining sterile technique during surgery. No device or router failure was experienced during any of the surgeries with no instances of re-calibration of the equipment.

**Figure 1:**
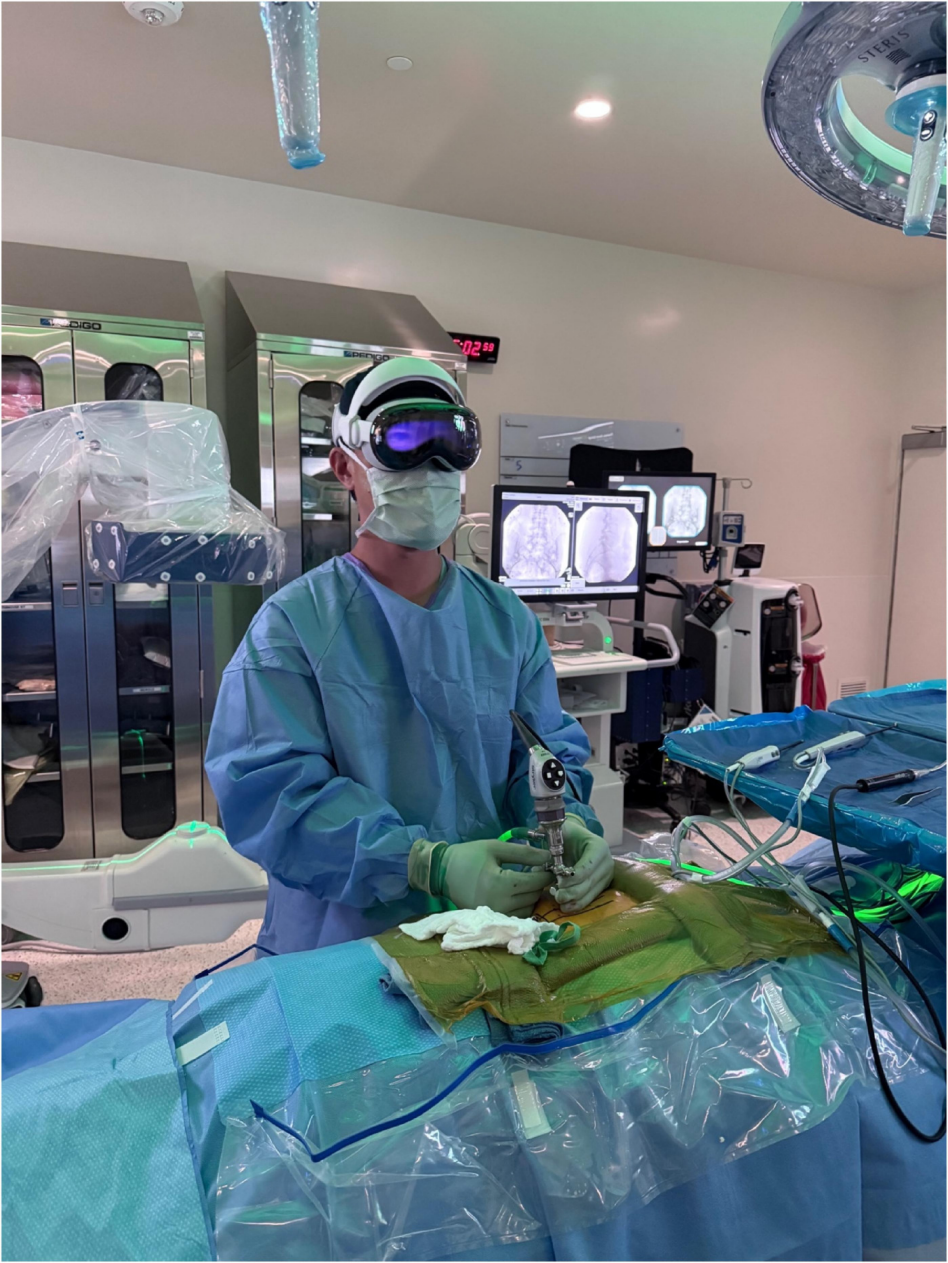
Intraoperative photograph depicting the AVP HMD used during surgery after the endoscope was placed into the viewing portal.

**Figure 2:**
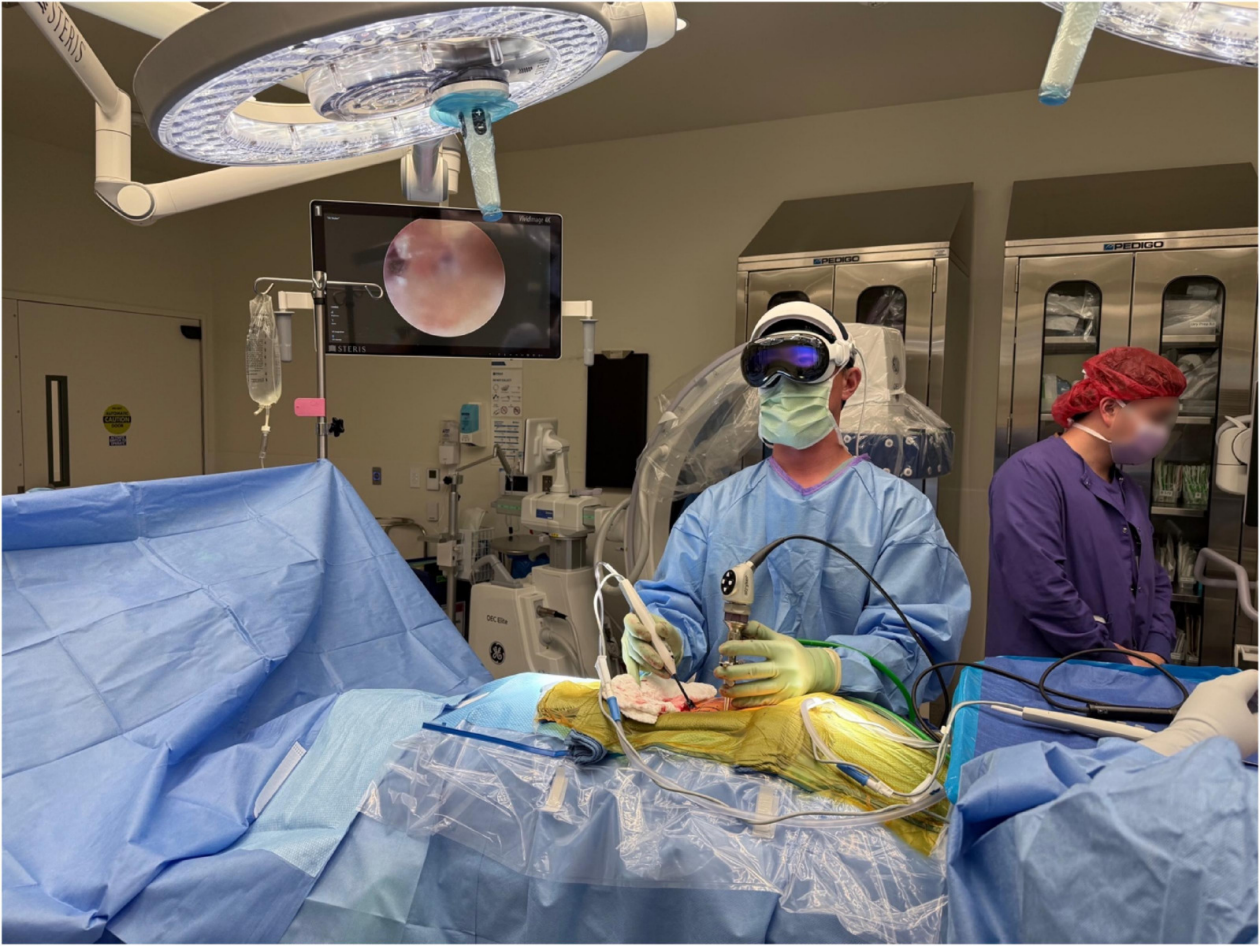
Intraoperative photograph depicting the AVP HMD used during surgery after placing the surgical instrument into the working portal.

**Figure 3:**
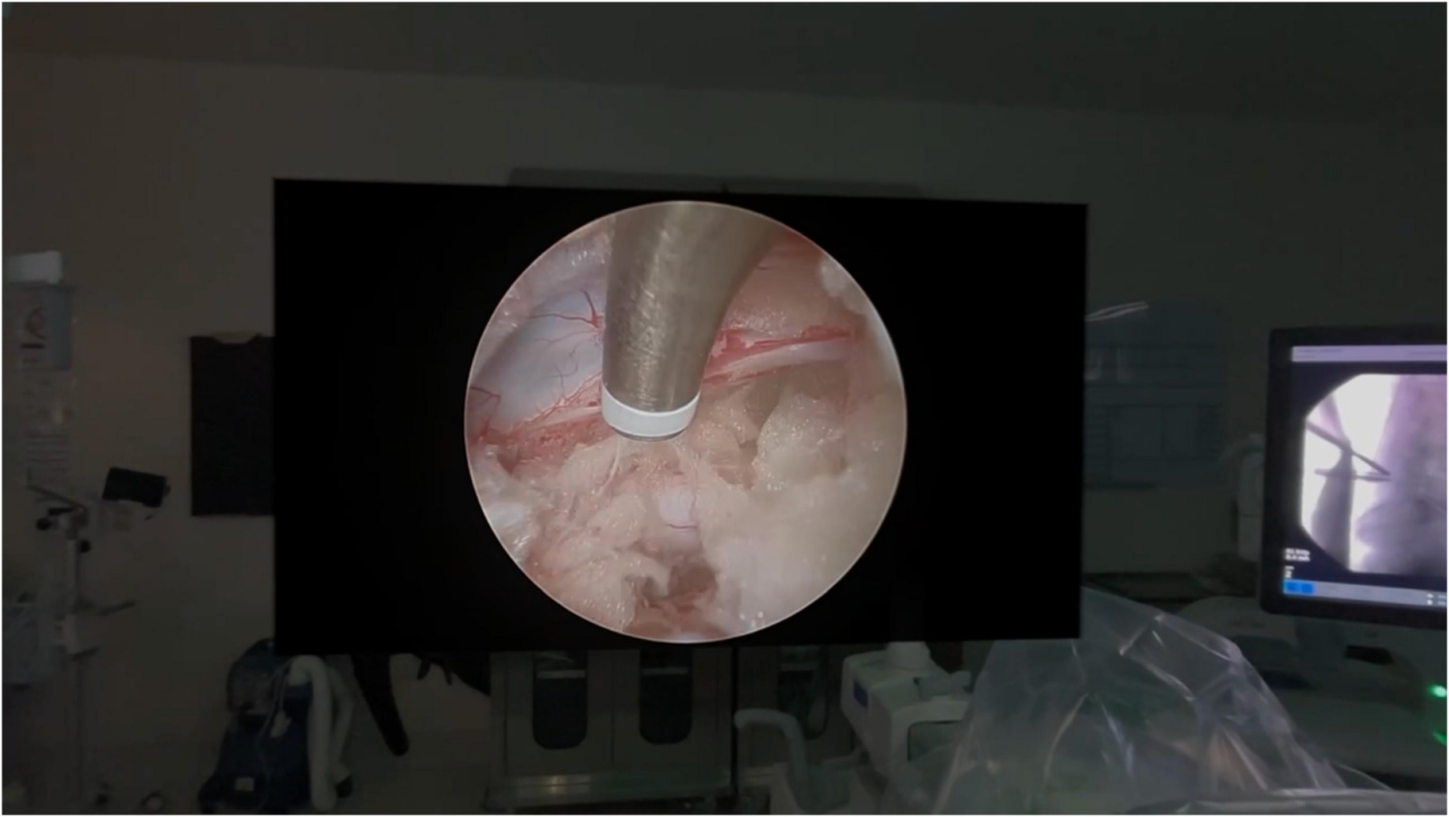
Intraoperative view of the large format endoscopic video digital projection superimposed onto the real environment as seen through the AVP HMD.

At this point, standard viewing and working portals were created to perform the biportal endoscopic procedure as previously described (Figures 1, 2) (5). Following insertion of the endoscopic camera through the viewing portal, the live endoscopic video feed was transmitted wirelessly to the AVP HMD via the dedicated Wi-Fi router. The biportal endoscopic laminotomy, decompression, and/or discectomy was then performed in standard fashion using the AVP HMD to visualize the endoscopic video rather than the operating room monitors (Figure 3) (17).

Of note, the AVP HMD was obtained by the senior author using independent research funding. Apple, Inc. had no role in device provision, study design, data analysis, or manuscript preparation.

### Statistical analysis

Univariate analysis was first performed to assess patient demographics and characteristics. Continuous variables were reported as means with standard deviations, and categorical variables as frequencies and percentages. Changes in patient-reported outcomes from preoperative baseline to postoperative follow-up were initially assessed for normal data distribution using the Shapiro–Wilk test as shown in Appendix 1. Normally distributed differences were defined as *p* > 0.05 and paired t-tests were subsequently employed to evaluate for statistical significance. For variables that did not meet normality assumptions (*p* < 0.05), a Wilcoxon signed-rank test was used as the nonparametric alternative. Visual representation of normality was additionally performed using quantile-quantile (Q-Q) plots as further supplementation to the Shapiro–Wilk results.

All statistical analyses were conducted with Stata Version 18.0 (StataCorp, 2019, Stata Statistical Software, College Station, Texas, USA) (21).

## Results

### Patient population

A total of 40 consecutive patients met the inclusion criteria for the study cohort utilizing the AVP HMD. The mean age of the population was 62.78 ± 16.12 years, with an average BMI of 27.90 ± 5.86, and 47.5% being female (Table 1). Lumbar stenosis was the predominant diagnosis (97.5%), followed by disc herniation (2.5%) (Table 1). Average operative time was 96.05 min ± 22.52 min. Across the cohort, unilateral laminotomy, bilateral decompression (ULBD) accounted for 37 cases (92.5%), unilateral lateral recess decompression for 2 cases (5%), and one patient (2.5%) underwent a discectomy (Table 1). The most frequently operated lumbar level was L4–5 (65%), with L3–4 and L5-S1 representing the next most frequent levels at 15%, respectively (Table 1). There were two incidental durotomies in the cohort, which were successfully repaired intraoperatively using collagen patches. There were no clinical sequelae as a result of the incidental durotomies. There were no other perioperative complications in this cohort.

**Table 1:** Perioperative outcome characteristics.

Variable	(*n* = 40) Mean + SD
Age	62.78 (16.12)
Sex (F)	19 (47.5%)
BMI	27.90 (5.86)
Diagnosis of Lumbar Stenosis	39 (97.5%)
Diagnosis of Lumbar Disc Herniation	1 (2.5%)
Operative Time	96.05 min (22.52)
Dural Tears	2 (5.0%)
SURG-TLX Score	22.24 (7.46)
Unilateral Laminotomy, Bilateral	37 (92.5%)
Decompression	2 (5%)
Unilateral Lateral Recess Decompression Discectomy	1 (2.5%)
L1–2 Operative Level	1 (2.5%)
L2–3 Operative Level	5 (12.5%)
L3–4 Operative Level	6 (15%)
L4–5 Operative Level	26 (65%)
L5–S1 Operative Level	6 (15%)
Preoperative VAS Back	5.40 (3.26)
Preoperative VAS Leg	6.85 (2.43)
Preoperative ODI	44.0 (18.67)
2 Week Postoperative VAS Back	2.12 (2.35)
2 Week Postoperative VAS Leg	1.72 (2.49)
2 Week Postoperative ODI	26 (18.88)
6 Week Postoperative VAS Back	1.81 (2.21)
6 Week Postoperative VAS Leg	1.38 (2.03)
6 Week Postoperative ODI	21 (18.29)
3 Month Postoperative VAS Back	2.71 (3.29)
3 Month Postoperative VAS Leg	2.11 (3.19)
3 Month Postoperative ODI	21 (22.74)

### SURG-TLX average

The cognitive workload assessment recorded via the SURG-TLX showed a mean global workload score of 22.24 ± 7.26 for cases utilized by the AVP HMD. This score depicts the self-perceived cognitive demand of the operative surgeon during these procedures.

### PRO analysis

**Figure 4:**
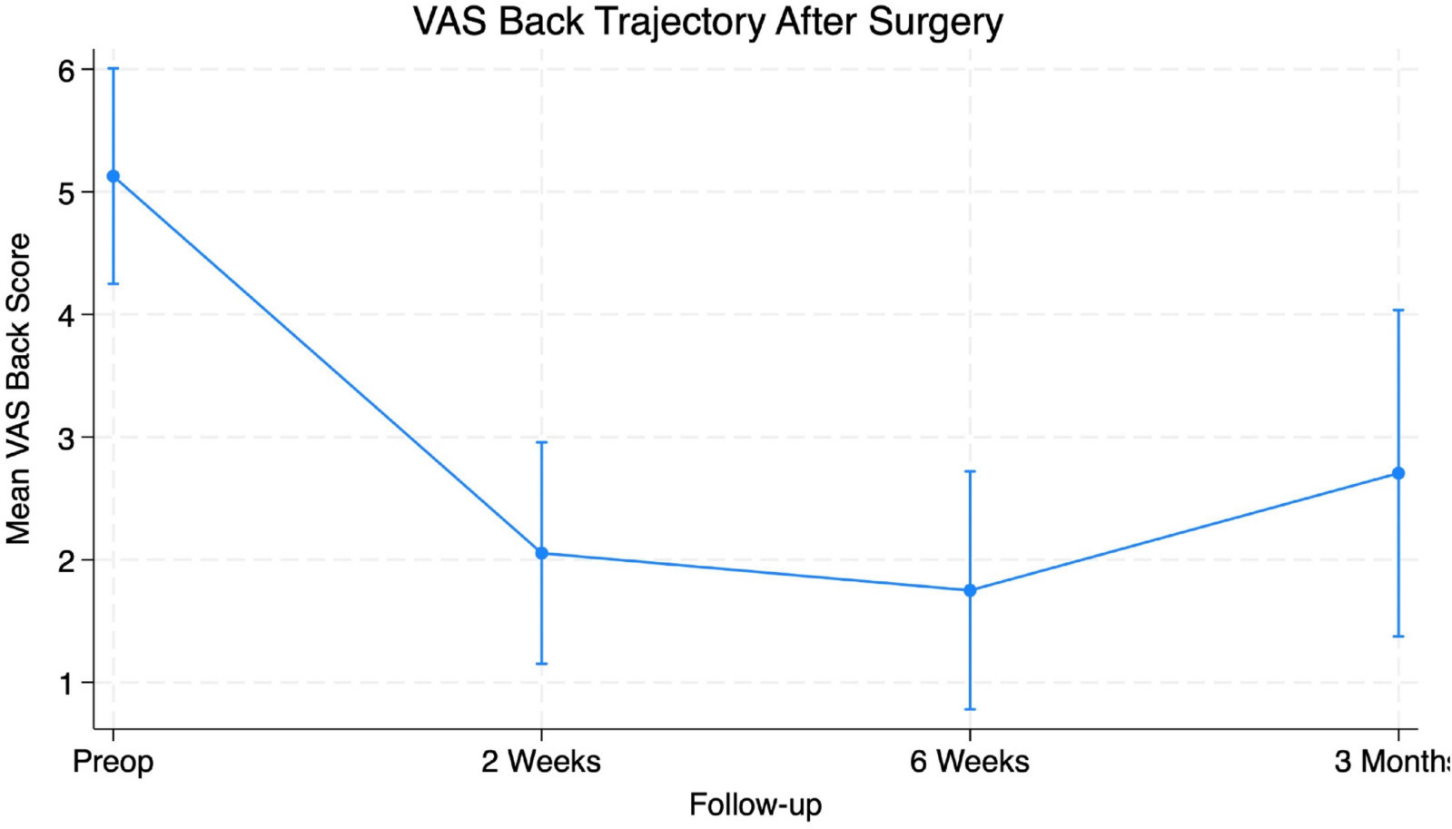
Figure depicting significant reductions in VAS back scores at all time points from preoperative to 3 months post-operatively (*p* < 0.05).

**Figure 5:**
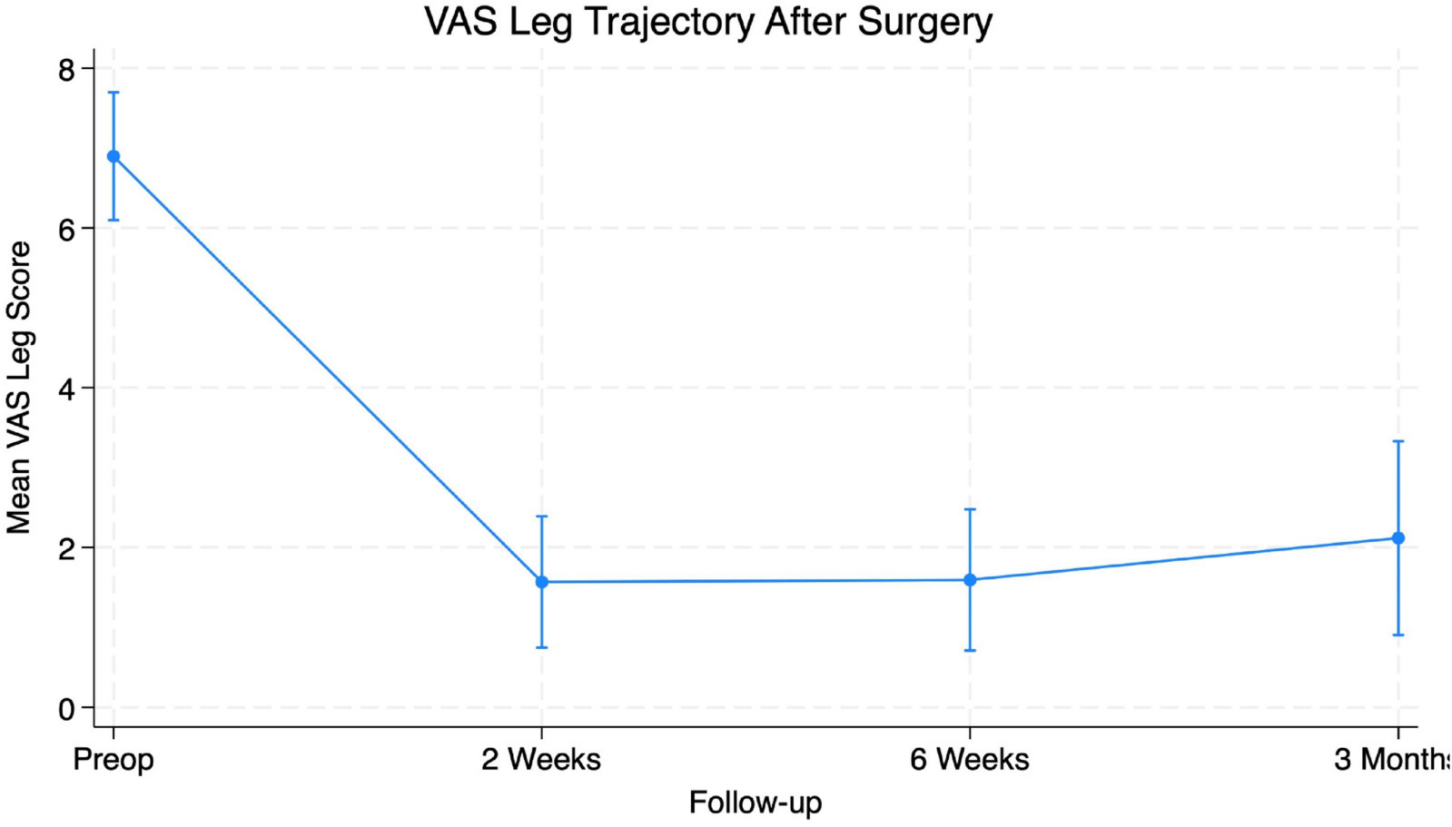
Figure depicting significant reductions in VAS leg scores at all time points from preoperative to 3 months post-operatively (*p* < 0.05).

**Figure 6:**
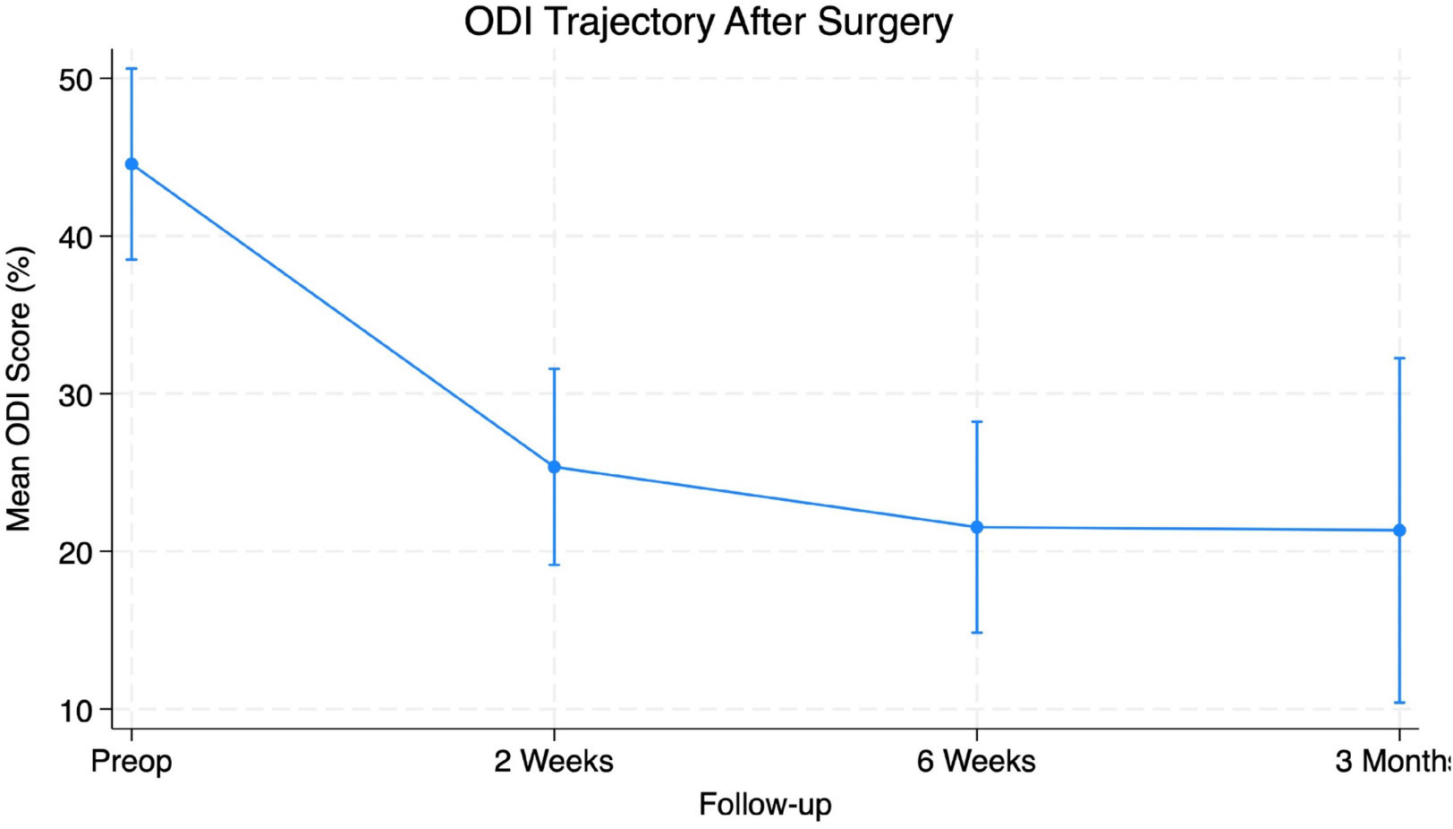
Figure depicting significant reductions in ODI scores at all time points from preoperative to 3 months post-operatively (*p* < 0.05).

Preoperative symptom burden was notable with a mean VAS Back score of 5.40 ± 3.26 and VAS Leg scores averaging 6.85 ± 2.43 (Table 1). Functional disability prior to surgery measured using the ODI score averaged 44.0% ± 18.67%. Two-week postoperative follow-up VAS Back scores decreased to 2.12 ± 2.35 and VAS Leg decreased to 1.72 ± 2.49. Additionally, ODI scores improved to a mean of 26% ± 18.88%. Six-week postoperative visit values were collected with VAS Back of 1.81 ± 2.21, Vas Leg of 1.38 ± 2.03, and ODI of 21.0% ± 18.29%. Patients demonstrated significant improvement in VAS Back (95% CI 2.40–4.97, *p* < 0.001), VAS Leg (95% CI 2.76–5.31, *p* < 0.001) and ODI (95% CI 16.55–30.26, *p* < 0.001) scores at six weeks after surgery (Table 2). At the three-month follow-up, VAS Back decreased to 2.71 ± 3.29, VAS Leg decreased to 2.12 ± 3.19, and ODI decreased to 21% ± 22.74%. Patients demonstrated significant improvement in VAS Back (95% CI 0.589–5.294, *p* = 0.017), VAS Leg (95% CI 2.547–6.629, *p* < 0.001) and ODI (95% CI 8.65%–44.01%, *p* = 0.007) scores at three months after surgery (Table 3). The PROs all demonstrated a significant decrease from preoperative levels (Figures 4–6).

**Table 2:** Pre-operative PRO versus post-operative six-week PRO paired *T*-test.

Pair	Comparison	Mean	Std. Deviation	Std. Error Mean	95% CI Lower	95% CI Upper	t	df	One-Sided p	Two-Sided p
Pair 1	VAS Back/Neck (Preop)—VAS Back/Neck (Postop)	3.688	3.56	0.629	2.404	4.971	5.86	31	<0.001	<0.001
Pair 2	VAS Leg (Preop)—VAS Leg (Postop)	4.063	3.601	0.636	2.764	5.361	6.383	31	<0.001	<0.001
Pair 3	ODI (Preop)—ODI (Postop)	23.419	18.654	3.35	16.577	30	6.99	30	<0.001	<0.001

**Table 3:** Pre-operative PRO versus post-operative 3-month PRO paired *T*-test.

Pair	Comparison	Mean	Std. Deviation	Std. Error Mean	95% CI Lower	95% CI Upper	t	df	One-Sided p	Two-Sided p
Pair 1	VAS Back/Neck (Preop)—VAS Back/Neck (Postop)	2.941	4.575	1.11	0.589	5.294	2.65	16	0.009	0.017
Pair 2	VAS Leg (Preop)—VAS Leg (Postop)	4.588	3.97	0.963	2.547	6.629	4.766	16	<0.001	<0.001
Pair 3	ODI (Preop)—ODI (Postop)	26.333	27.828	8.033	8.652	44.015	3.278	11	0.004	0.007

## Discussion

At present, there is very little in the published literature investigating the use of MR technology such as the AVP HMD in endoscopic spine surgery. A recent study by van Isseldyk et al., investigated the use of an augmented reality headset in uniportal endoscopic spine surgery and evaluated the headset with the NASA-TLX tool with 10 surgeons performing surgery with the headset and compared it to a standard monitor (19). They found that the augmented reality headset had lower NASA-TLX scores and improved ergonomics using the Rapid Upper Limb Assessment (RULA). The authors used the raw NASA-TLX score rather than the weighted score, which makes comparison difficult with our present study. NASA-TLX was originally designed to evaluate how humans interact with complex machinery such as in aviation. SURG-TLX is an adaptation of the NASA-TLX and designed specifically for the operating room environment by replacing the dimensions of Performance for Task Complexity, Effort for Situational Stress, and Frustration for Distractions, which may be more relevant in the surgical environment. Due to this, we used the SURG-TLX in our study to evaluate the use of the AVP HMD in biportal endoscopic spine surgery.

In this study, the average SURG-TLX Score was 22.24 ± 7.46, which reflects a relatively low cognitive burden on the operating surgeon. This grading system aligns with the lower spectrum of the 0–100 workload scale previously reported in literature (20). The low SURG-TLX score observed suggests that the MR interface did not increase the perceived demands on the operating surgeon. In previously published studies, a cognitive score of 50–55 has potential to be linked to increased performance errors, further supporting that the present scores fall well below the risk thresholds described in other surgical settings (20). The vast majority of the cases in this series consisted of ULBD cases with 2 lateral recess decompressions and 1 discectomies. In our experience, the utility of the AVP HMD did not differ with various pathology or casew complexity since the MR headset was utilized as a visualization tool. The 4 K video virtual projection in the AVP HMD appears 2–3 times larger than the standard operating room monitor to the operating surgeon. This assists the surgeon to visualize the endoscopic video with tremendous detail. However, this improvement in visualization is subjective and is not supported with an objective validated visualization measure in this study.

This study demonstrated a low complication rate with an incidental durotomy rate that is comparable to published rates in traditional endoscopic spine procedures (18). The durotomies were not influenced by the AVP HMD as they were associated with dural adhesions to the hypertrophied ligamentum flavum, which is commonly seen in the senior surgeon's practice irregardless of the AVP HMD. Furthermore, patient PROs including VAS Back, VAS Leg, and ODI showed statistically significant improvement with early functional recovery up to three months after surgery, which are characteristic of the biportal endoscopic technique itself and not the AVP HMD. This highlights that MR-assisted surgeries did not hinder operative performance or reduce clinical success. These findings suggest that the AVP HMD can be utilized during endoscopic spine surgery with no increased risk to the patient and highlight the usability of the AVP HMD intraoperatively.

This study has multiple limitations that should be considered. This study was designed to be a feasibility study to demonstrate the implementation of the AVP HMD in biportal endoscopic spine surgery. However, there was no comparison cohort designed into the study evaluating standard monitors, which is major limitation. In addition, objective ergonomic or performance metrics were not collected in the study as it was not designed into the study. The purpose of this study was to demonstrate the clinical feasibility of using the AVP HMD in endoscopic spine surgery and that utilizing the AVP HMD did not increase the surgeon's perceived workload. From the senior surgeon's perspective, the ergonomic impact of the AVP HMD was minimal with no instances of head or neck pain or fatigue and no restrictions of surgeon movement or surgical posture. The ergonomics is improved by adding a headpiece to the AVP HMD that shifts the weight from the front of the face to the top of the head, improving the comfort and wearability of the headset for longer periods of time (Figures 1, 2). However, future research studies incorporating objective ergonomic and performance data is currently being designed to further investigate this important aspect of these types of devices.

The small sample size limits this study's statistical power and generalizability. The follow up was short at three months after surgery but the long-term clinical results of biportal endoscopic spine surgery is well documented in the literature. Additionally, only the AVP HMD was utilized in this study and was not compared to other MR platforms, although there are very few commercially available MR HMD systems at this time. The use of the SURG-TLX survey, while validated, only assesses six subjective domains and could be expanded allowing for further evaluation of ergonomic and usability features. The surgeon completed the SURG-TLX survey immediately after the surgery, which can reduce the recall bias of the study. However, desirability bias by the surgeon may come into play, which can affect the SURG-TLX results. In addition, trainees were not included in this study, which is a major limitation of the study since the workload may differ based on surgical experience. Surgeon experience contributes to lower cognitive workload as residents consistently demonstrate higher SURG-TLX scores compared to their more experienced counterparts (22). Future study is necessary to compare the SURG-TLX results of trainees and experienced surgeons while using the AVP HMD. Also, future prospective studies with larger sample sizes, longer-follow-up periods, as well as more comparison of the AVP HMD to other MR platforms are necessary to further characterize the role of MR technology in endoscopic spine surgery.

## Conclusion

This is an early feasibility study describing the incorporation of MR technology during biportal endoscopic lumbar surgery. We utilized the SURG-TLX, a validated cognitive workload measurement to demonstrate that using the AVP HMD did not increase the cognitive burden of the surgeon during surgery. Additionally, using the AVP HMD did not adversely affect clinical outcomes or increase the risk of perioperative complications. As MR technology continue to advance, it holds promise for incorporation in endoscopic spine surgery in the future.

## Data Availability

The raw data supporting the conclusions of this article will be made available by the authors, without undue reservation.

## Appendix 1. Shapiro–Wilk *W* Test to test for normal data distribution.

**Table:**

Variable	Shapiro–Wilk W Test
6 Week Change in VAS Back	0.34017
6 Week Change in VAS Leg	0.00789
6 Week Change in ODI	0.64910
3 Month Change in VAS Back	0.94443
3 Month Change in VAS Leg	0.67400
3 Month Change in ODI	0.47222
